# A patient with superior mesenteric artery lymph node metastasis after laparoscopic gastrectomy for gastric cancer with Adachi type VI vascular anomaly: A case report

**DOI:** 10.1016/j.ijscr.2020.11.086

**Published:** 2020-11-24

**Authors:** Kenjiro Hirai, Taro Aoyama, Wataru Hirata, Hiroshi Okabe, Akira Mitsuyoshi

**Affiliations:** a Department of Surgery, Otsu City Hospital 2-9-9 Motomiya, Otsu City, Shiga, 520-0804, Japan; b Department of Surgery, Graduate School of Medicine, Kyoto University 54 Shogoin, Kawahara-cho, Sakyo-ku, Kyoto City, 606-8507 Japan; c Department of Gastroenterological Surgery, New Tokyo Hospital 1271, Wanagaya, Matsudo City, Chiba, 270-2232, Japan

**Keywords:** Adachi type VI, Superior mesenteric artery lymph node metastasis, Laparoscopic gastrectomy

## Abstract

**Introduction:**

Due to recent advances in surgical procedures and instruments, laparoscopic gastrectomy for gastric cancer has been widely performed, and previous studies reported laparoscopic surgery for gastric cancer with Adachi type VI vascular anomaly. In Adachi type VI patients, the common hepatic artery (CHA) originates from the superior mesenteric artery (SMA); therefore, the route of lymph flow differs from the normal route, and the supra- and infrapyloric lymph nodes (LN) may reach SMA LN. However, metastasis has not yet been reported. A case of SMA LN metastasis 3 years after laparoscopic distal gastrectomy for gastric cancer with Adachi type VI CHA anomaly, which was diagnosed using preoperative computed tomography (CT), was described herein.

**Presentation of case:**

The patient was a 77-year-old male. Laparoscopic distal gastrectomy and D2 + 14v LN dissection for gastric cancer with Adachi type VI vascular anomaly were performed. Three years after surgery, periodic CT revealed swelling of regional and mediastinal SMA LN, leading to a diagnosis of recurrent gastric cancer. A histopathological examination of the resected specimen showed metastases to the greater curvature right group and infrapyloric LN.

**Discussion:**

Metastasis to LN No. 6 may have reached SMA LN via the gastroduodenal artery and CHA, but not the celiac artery.

**Conclusion:**

If preoperative diagnostic imaging suggests metastasis to the greater curvature right group and pyloric regions in gastric cancer patients with Adachi type VI vascular anomaly, LN dissection along CHA originating from SMA and the hepatomesenteric trunk needs to be considered.

## Highlights

• SMA LN metastasis after laparoscopic surgery for gastric cancer with Adachi type VI.
• In Adachi type VI, the greater curvature and peripyloric LN may reach SMA via CHA.
• LN dissection along CHA and the hepatomesenteric trunk needs to be considered.

## 1 Introduction

Due to recent advances in surgical procedures and instruments, laparoscopic surgery for gastric cancer has been widely performed [1], and previous studies reported laparoscopic surgery for gastric cancer with Adachi type VI vascular anomaly [2,3]. In group 2 lymph node (LN) dissection (D2) during gastrectomy for gastric cancer, the pancreatic capsule needs to be incised at the upper margin of the pancreas, followed by exposure of the common hepatic and splenic arteries. However, since the common hepatic artery (CHA) is absent at the upper margin of the pancreas in patients with Adachi type VI, caution is needed. CHA originates from the superior mesenteric artery (SMA) in these patients; therefore, the route of lymph flow differs from the normal route, and the greater curvature right group and supra-/infrapyloric LN may reach SMA LN via CHA [4,5]. A patient who underwent laparoscopic distal gastrectomy with D2 + 14v dissection for gastric cancer with Adachi type VI, which was preoperatively diagnosed using three-dimensional computed tomography (3D-CT) angiography, was described herein. Common hepatic and SMA LN metastases and cancerous pleuritis developed after surgery, leading to a diagnosis of recurrent gastric cancer. In the present case, a histopathological examination of the resected specimen revealed metastases to the greater curvature right group and infrapyloric LN, suggesting metastasis from infrapyloric LN to SMA LN, but not the celiac artery. This is the first case of SMA LN metastasis after surgery for gastric cancer with Adachi type VI. This work has been reported in line with the SCARE criteria [6].

## 2 Presentation of case

**Fig. 1:**
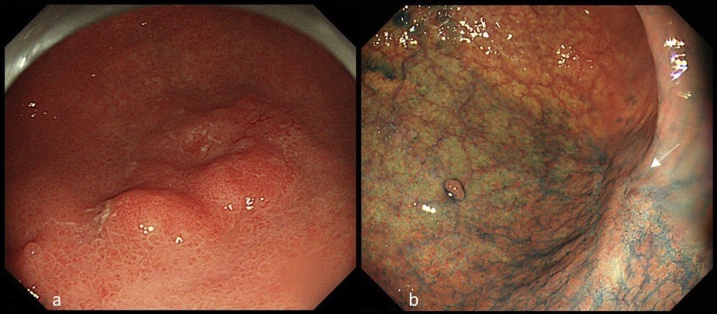
Upper gastrointestinal endoscopy. a) In 2011, a 0-IIa lesion of the greater curvature at the gastric angle was detected, and ESD was performed. b) In 2016, there was no relapse at the site of the post-ESD ulcerative scar (arrow).

**Fig. 2:**
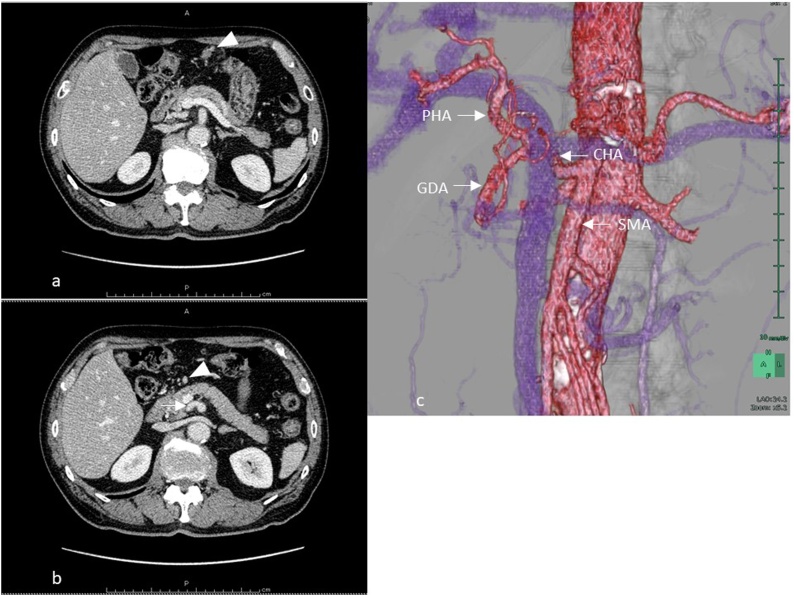
CT findings. a) Greater curvature right group lymph node swelling was observed (arrowhead). b) Infrapyloric lymph node swelling was noted (arrowhead). The common hepatic artery (CHA) had directly branched from the superior mesenteric artery (SMA)(arrow) and there was no artery on the ventral side of the portal vein. c) Vascular rearrangement images also showed similar findings, with Adachi type VI vascular anomaly. SMA: superior mesenteric artery, CHA: common hepatic artery, PHA: proper hepatic artery, GDA: gastroduodenal artery.

A 77-year-old male presented with LN metastasis after endoscopic submucosal dissection (ESD). There was no family history. He previously underwent surgery for a right inguinal hernia and had diabetes mellitus. He was regularly taking a hypoglycaemic drug. In 2011, ESD of type 0-IIa, tub2 > tub1 of the greater curvature at the gastric angle, which was detected on screening, was performed (Fig. 1a). A pathological examination revealed tub2 > tub1, T1b (SM1 400 μm), ly (+), v (−), VM0, and HM0. In 2016, there was no relapse at the site of the post-ESD ulcerative scar, and biopsy did not show malignancy (Fig. 1b). Follow-up contrast-enhanced CT revealed greater curvature right group and infrapyloric LN swelling, suggesting LN metastasis from gastric cancer (Fig. 2a and b). 3D angiography indicated Adachi type VI involving CHA branching from SMA (Fig. 2c). Based on these findings, the patient was diagnosed with post-ESD LN metastasis from gastric cancer (TXN1M0), and laparoscopic distal gastrectomy with D2 + 14v dissection was performed.

**Fig. 3:**
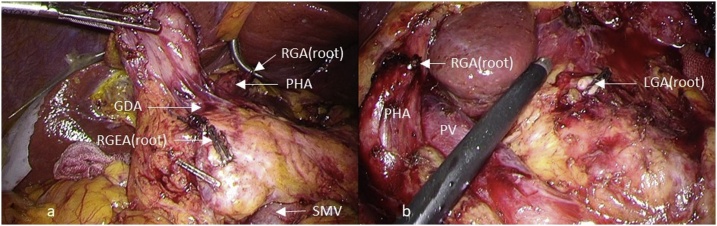
Intraoperative findings: After the completion of dissection. LGA: left gastric artery, RGA: right gastric artery, RGEA: right gastroepiploic artery, PV: portal vein, SMV: superior mesenteric vein.

In LN No. 8a dissection, the portal vein was exposed at the upper margin of the pancreatic body, and dissection was performed at the anterior layer of the portal vein. Tissue involving LN at the upper margin of the pancreas was dissected to the level of the right crus of the diaphragm on the cephalic side, and this was resected to the left to dissect LN No. 8a. After confirming the splenic artery, the peripheries of the celiac artery and left gastric artery were dissected. After LN dissection, a specimen was extirpated, and Billroth II reconstruction was performed. The operative time was 359 min and the volume of blood loss was 20 mL (Fig. 3). Histopathologically, tub1 at the site of the post-ESD scar, T3 (SS), N2 (No. 4d 3/6 nodes, No. 6 3/4 nodes), ly0, and v0 were suggested.

**Fig. 4:**
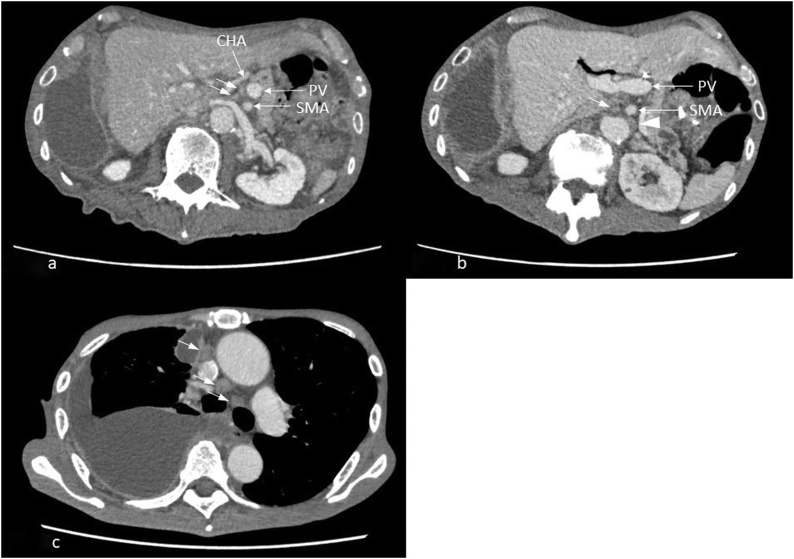
CT findings. a) Lymph node swelling was observed along the common hepatic artery (arrow). b) Superior mesenteric artery root (arrow) and paraaortic (arrowhead) lymph node swelling was noted. c) Right pleural effusion retention and swelling of multiple mediastinal lymph nodes were observed (arrow).

After postoperative adjuvant chemotherapy (S-1 was administered orally at a dose of 70 mg/m^2^ daily for the first 4 weeks of a 6-week cycle for 12months), the follow-up was continued. Periodic contrast-enhanced CT in 2019 revealed multiple pulmonary nodes with pleural effusion and mediastinal LN swelling, in addition to CHA and SMA LN swelling, leading to a diagnosis of recurrent gastric cancer. Chemotherapy (S-1 was administered orally at a dose of 70 mg/m^2^ daily for the first 4 weeks of a 6-week cycle, followed by ramucirumab 8 mg/kg intravenously on days 1 and 15, plus paclitaxel 80 mg/m^2^ intravenously on days 1, 8, and 15 of a 28-day cycle) was performed; however, the patient died of gastric cancer five months later (Fig. 4).

## 3 Discussion

Accurate and safe LN dissection is necessary when performing surgery for gastric cancer because the incidence of LN metastasis at the upper margin of the pancreas is high. Furthermore, a detailed understanding of the courses of the celiac artery branches is needed [7]. In Adachi’s classification, CHA, splenic artery, and left gastric artery branching from the celiac artery are classified into types I to VI and Groups 1–28 [8]. Type VI refers to a condition in which the course of CHA branching from SMA does not involve the upper margin of the pancreas or ventral side of the portal vein. Its incidence is 2.0% according to Adachi.

Caution is needed for LN No. 8a dissection in patients with Adachi type VI. The dorsal margin of dissection is established as the portal vein, not as CHA, and dissection is performed by regarding this area as No. 8a [3,9]. In the present case, the right margin of LN No. 8a dissection was established based on the courses of the gastroduodenal and proper hepatic arteries. The anterior surface of the portal vein was subsequently exposed, and LN No. 8a dissection was performed while preserving this layer. LN No. 8a dissection after preoperative detailed vascular course assessments using 3D angiography with careful intraoperative observations facilitate laparoscopic gastrectomy with accurate and safe D2 dissection, even on Adachi type VI patients.

The route of LN metastasis may change in Adachi type VI patients. Previous studies suggested that gastric cancer with Adachi type VI metastasizes to SMA LN; however, metastases have not yet been reported [4,5]. The present case was pathologically diagnosed with LN No. 6 and 4d metastases. There are 3 lymph flow routes for LN No. 6: a route in which the celiac artery is reached through the gastroduodenal and common hepatic arteries, a route in which the right margin of the superior mesenteric vein is reached via the right gastroepiploic vein to the gastrocolic trunk, and a route in which the splenic artery trunk or postpancreatic LN are reached. In the Gastric Cancer Treatment Guidelines, typical dissection is recommended to involve LN Nos. 9 and 14v [10,11]. The lymph flow route that applies to LN No. 6 needs to be assessed in reference to its subclassification proposed by Shinohara et al. [12,13]. Briefly, LN No. 6 is anatomically divided into 3 regions: LN No. 6a involving the right gastroepiploic artery root to the 1st vessel of the greater curvature (dorsal mesogastrium), LN No. 6v involving the periphery of the right gastroepiploic vein (mesoduodenum), and LN No. 6i involving the infrapyloric mesentery consisting of the infrapyloric artery/vein. The incidence rates of No. 6a, 6v, and 6i LN metastases in the M region of > T2 patients, such as the present case, are reportedly 6.4, 2.1, and 2.1%, respectively [14]. In the present case, LN No. 6 was not classified according to the above subclassification when the pathological specimen was submitted, and the following explanation is a matter of speculation. There was no metastasis to LN No. 14v, whereas metastasis to LN No. 6a corresponding to the dorsal mesogastrium, similar to LN No. 4d to which cancer had metastasized, was suspected. Briefly, among infrapyloric LN, metastasis to LN No. 6a may have reached SMA LN via the gastroduodenal artery, but not the celiac artery.

McDonald et al. proposed the following 3 routes of pulmonary metastasis from gastric cancer: (1) hematogenous metastasis to the lungs through the inferior vena cava via the portal vein and liver from the gastric vein, (2) lymphogenous metastasis to the lungs through the superior vena cava via the chyle cistern and thoracic duct from the regional LN, and (3) disseminated metastasis to the lungs via the diaphragm and thoracic cavity [15]. In the present case, systemic disease was present at the time of relapse; however, CT did not reveal liver metastasis, peritoneal nodes, or ascites. Therefore, lymphogenous metastasis from SMA LN, which is a regional LN in Adachi type VI patients, was suspected as the main mode of pulmonary invasion.

Since SMA LN metastasis from gastric cancer with Adachi type VI has not yet been reported, the present case may be valuable. An area corresponding to LN No. 8a, which was matched to normal lymph flow, and LN Nos. 9 and 14v were dissected. However, in Adachi type VI patients, LN No. 8a may be CHA originating from SMA and LN No. 9 may be the hepatomesenteric trunk. If preoperative diagnostic imaging suggests metastasis to the greater curvature right group or pyloric region, as demonstrated in the present case, LN dissection along CHA and the hepatomesenteric trunk as areas corresponding to LN Nos. 8a and 9 needs to be considered. Further studies are required to clarify whether SMA LN, which are not dissected during standard surgery for gastric cancer, need to be dissected in Adachi type VI patients.

## 4 Conclusion

A case of SMA LN metastasis after laparoscopic surgery for gastric cancer with Adachi type VI, which is relatively rare, was described herein. In surgery, the dorsal margin of LN No. 8a dissection was established as the portal vein, but not as CHA, and dissection was performed by regarding the area as No. 8a. When performing surgery for gastric cancer with Adachi type VI, lymph flow may differ from the normal route, and if preoperative diagnostic imaging suggests metastasis to the greater curvature right group or pyloric region, LN dissection along CHA originating from SMA and the hepatomesenteric trunk needs to be considered.

## Declaration of Competing Interest

The authors report no declarations of interest.

## Funding

No funding was obtained for this study.

## Ethical approval

The present study was conducted in accordance with the ethics of the Declaration of Helsinki and was approved by the Ethics Committee of Otsu City Hospital. (No32. Otsu City Hospital).

## Consent

Written informed consent was obtained from the patient for publication of this case report and accompanying images. A copy of the written consent is available for review by the Editor-in-Chief of this journal on request.

## Author contribution

Conception and design of the study: KH.

Performing surgery and attending to the patient postoperatively: KH, TA, WH, and HO.

Level of surgical experience: Operators were experts of gastrointestinal laparoscopic surgery.

Analysis and interpretation of data: KH, TA, and WH.

Drafting of the manuscript: KH, HO.

Review of the final version of the manuscript: TT, HO, and AM.

## Registration of research studies

Not applicable.

## Guarantor

Kenjiro Hirai.

Akira Mitsuyoshi.

## Provenance and peer review

Not commissioned, externally peer-reviewed.
